# Pictorial guide for variants of Covid-19: CT imaging and interpretation

**DOI:** 10.1259/bjro.20220011

**Published:** 2022-10-05

**Authors:** Giacomo Bonito, Valeria Martinelli, Francesco Vullo, Fabrizio Basilico, Eleonora Polito, Antonella Izzo, Laura Corso, Paolo Ricci

**Affiliations:** 1 Unit of Emergency Radiology, Policlinico Umberto I Hospital, Sapienza University of Rome, Viale Regina Elena, Rome, Italy; 2 Department of Radiological, Oncological and Pathological Sciences, Sapienza University of Rome, Viale Regina Elena, Rome, Italy

## Abstract

Typical radiologic images of Covid-19 pneumonia consists in a wide spectrum of chest manifestations, which range from peripheral predominant ground-glass opacities to an organizing pneumonia pattern, with additional features including crazy-paving, consolidations, fibrotic streaks and linear opacities.

With variants imaging profile of Covid-19 evolves, producing relatively atypical/indeterminate CT pattern of pulmonary involvement, which overlap with imaging features of a variety of other respiratory diseases, including infections, drug reaction and hypersensitivity pneumonia. Our knowledge of these radiological findings is incomplete and there is a need to strengthen the recognition of the many faces of Covid-19 pneumonia.

## Introduction

In March 2020, the World Health Organization (WHO) declared the coronavirus disease 2019 caused by Sars-CoV2 virus (COVID-19) as a pandemic and global public health emergency. This is the first pandemic in modern era presenting with unknown clinical syndrome and therapy: both of them were defined progressively, after the spread of the disease.

At first COVID-19 was interpreted as an infectious disease limited to the lungs, whereas only afterwards the vasculitic pathogenesis was described as affecting other organs. COVID-19 outcome and long-COVID syndrome have been described only recently.

Imaging features of COVID-19 were likewise progressively portrayed, after the widespread of the disease, leading to a new nosographic radiological entity.^1^

Additional aspects of this infectious disease are represented by the wave-shaped trend of the pandemic (“waves”, variably named, depending on the countries), and by the outbreak of new variants of the virus. Few of them were declared by the WHO “Variant of Concern”, given their enhanced transmissibility or virulence, and their potential to weaken the effectiveness of therapy and vaccination strategies. Five variants have been designated by the WHO:

α (B. 1.1.7), first described in UK in late December 2020;

β (B. 1.351), first reported in South Africa in December 2020;

γ (P.1), first described in Brazil in January 2021;

δ (B.1.617.2): first reported in India in December 2020 and the more recent one, the Omicron (B. 1.1.529), reported in South Africa in October 2021.^2^

δ variant was associated with increased viral load and prolonged viral shedding in respiratory samples compared to previous variants, with implications on infection control and public health policy: this leaded to higher rates of hospitalization, ICU admission and death. Many studies have shown that Omicron VOC have a less severe clinical presentation than the δ VOC.^3^

Although reference standard for diagnosis is RT-PCR, chest CT remains a complementary diagnostic tool limited to specific indications, playing a role in clinical assessment of patients with moderate to severe illness.^1^

This is a short pictorial guide showing the key imaging features of variants of COVID-19, which could be useful in daily radiological practice.

## CT protocol

CT is the gold-standard in diagnostic imaging of COVID-19 pneumonia.

Chest CT examination should be performed with high-resolution non-contrast technique with a reconstructed slice thickness of 1.0–1.5 mm.^1^

## Imaging findings

The subsequent waves of pandemic showed different and atypical chest CT features, that are less specific or relatively uncommon, and can occur in association with or even mimic a variety of infectious and non-infectious processes.^4^

### GGO with predominant upper lobes involvement and random distribution

**Figure 1.**
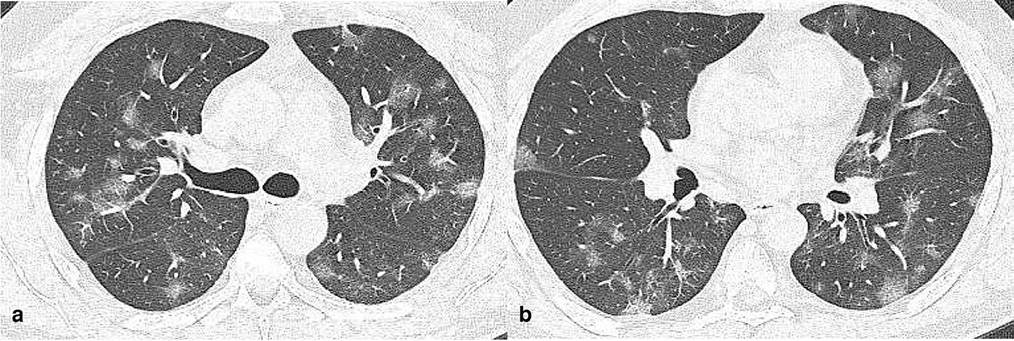
Axial CT images (**a, **b) of a 34-year-old male infected by SARS-CoV-2 α variant (3 days since symptoms onset). CT showed centrally and peripherally located multifocal GGO opacities with mainly involvement of upper lobes.

The typical Covid-19 started as bilateral ground-glass opacities (GGOs) in posterior segments of lower lobes with peripheral distribution, which then developed into the crazy paving appearance and subsequent consolidation.^5^ With variants, GGO may present random distribution (no predilection for subpleural or central regions), with prevalent/exclusive involvement of upper lobes (Figure 1).

### Single isolated involvement

**Figure 2.**
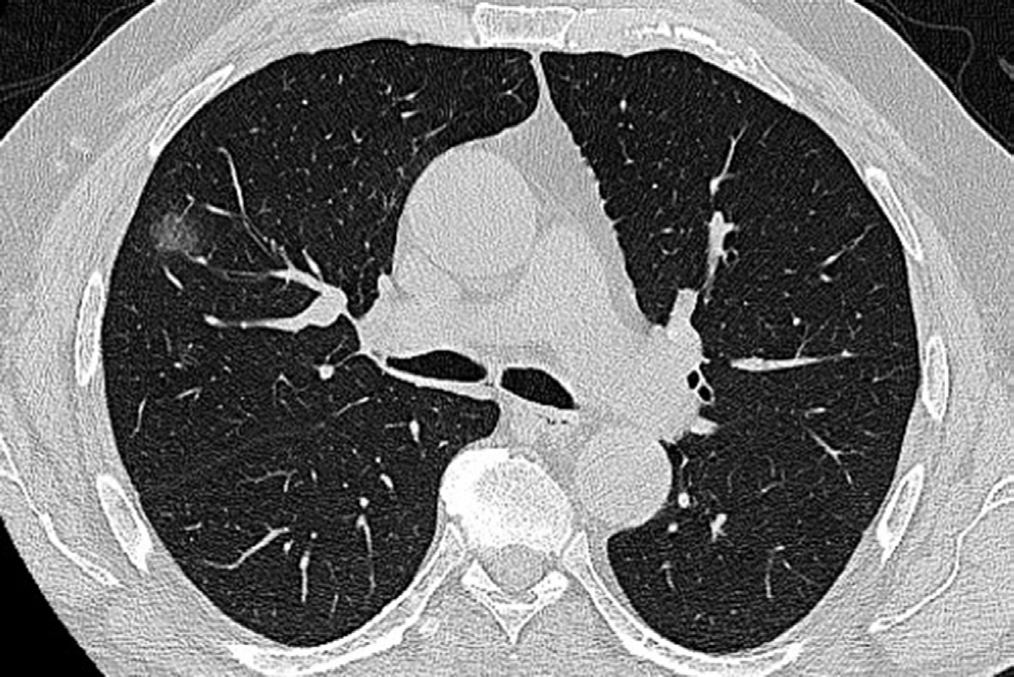
A 54-year-old female COVID −19 patient (2 days since symptoms onset) infected by Omicron variant. CT scan shows single ground glass opacity with rounded appearance, in the right upper lobe.

**Figure 3.**
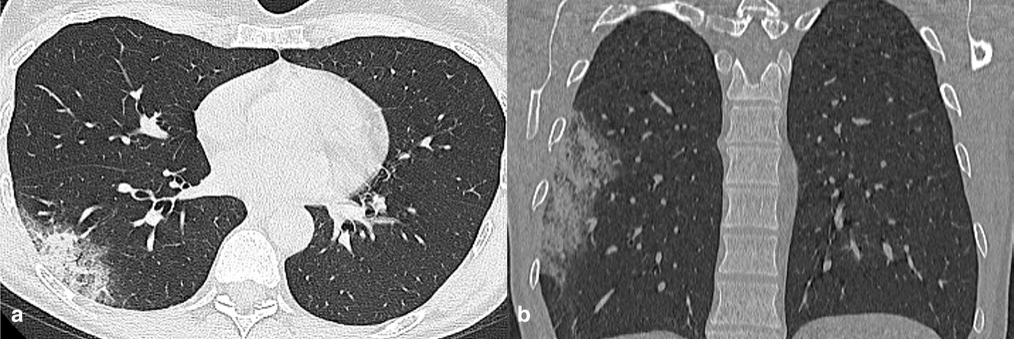
A 78-year-old male arriving at the emergency department with fever. Axial (a) and coronal reformatted (b) CT images documented a subpleural GGO in right lower lobe. Notably, there were signs of consolidation in the context of the lesion. The real-time reverse transcription polymerase chain reaction test was positive for Sars-Cov-2 infection. Subsequent genetic sequencing detected α variant. GGO, ground-glass opacity.

During third and fourth wave, we observed solitary involvement in any lobe, in the form of GGO or denser (Figures 2 and 3): in early stage of disease, if this sign is not accompanied by other features or patterns, like small areas of consolidation or interlobular septal thickening, alternative diagnoses should be considered. Crucial is the correlation with clinic and laboratory.

### Isolated lobar consolidation

Consolidations were described as patchy, multifocal or segmental in the late phase of the disease, during first wave of pandemic; they also represented a possible indicator of disease progression.^6^

With variants, we observed homogenous consolidations involving exclusively one lobe, with visible air-bronchogram sign.

**Figure 4.**
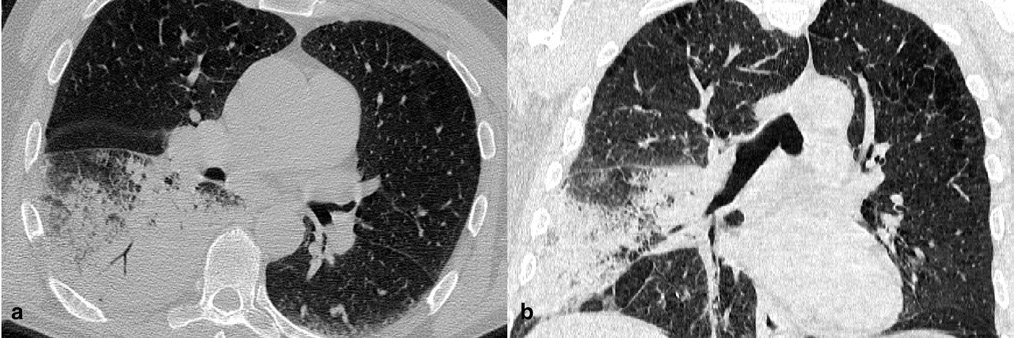
A 57-year-old COVID-19 patient (δ variant) presenting fever and dry cough for 3 days. Axial (a) and coronal (b) reformatted CT images showed pneumonia with isolated lobar consolidation in the right lower lobe.

This feature is not specific, since it could be caused by different infectious agents, like Pneumococcus or Legionella species (Figure 4).^7^

### Pulmonary target sign (bullseye sign)

**Figure 5.**
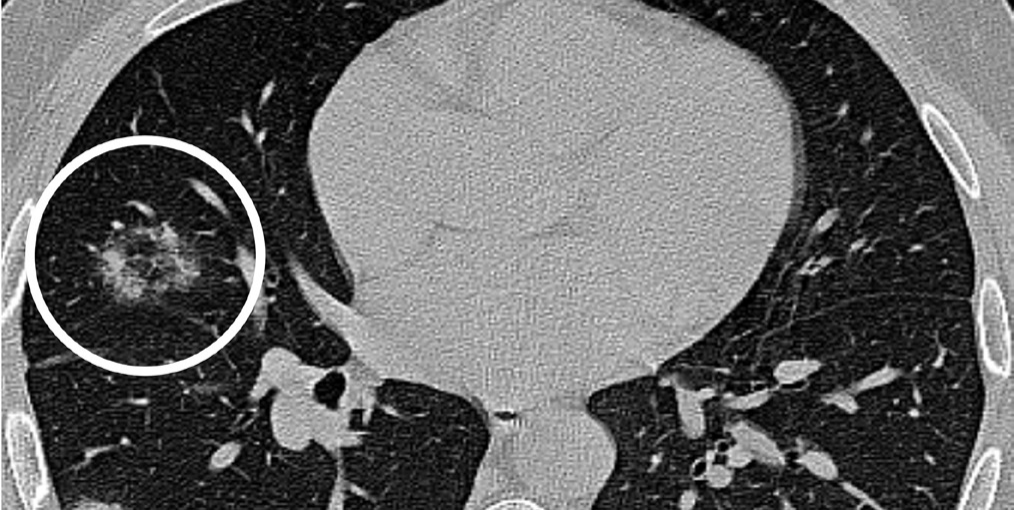
A 32-year-old female infected with COVID-19 (δ Variant): axial unenhanced CT image showed focus of central ground glass nodule surrounded by an inner ring of air and an outer ring of ground glass (white circle). This appearance ressembles a bullseye.

**Figure 6.**
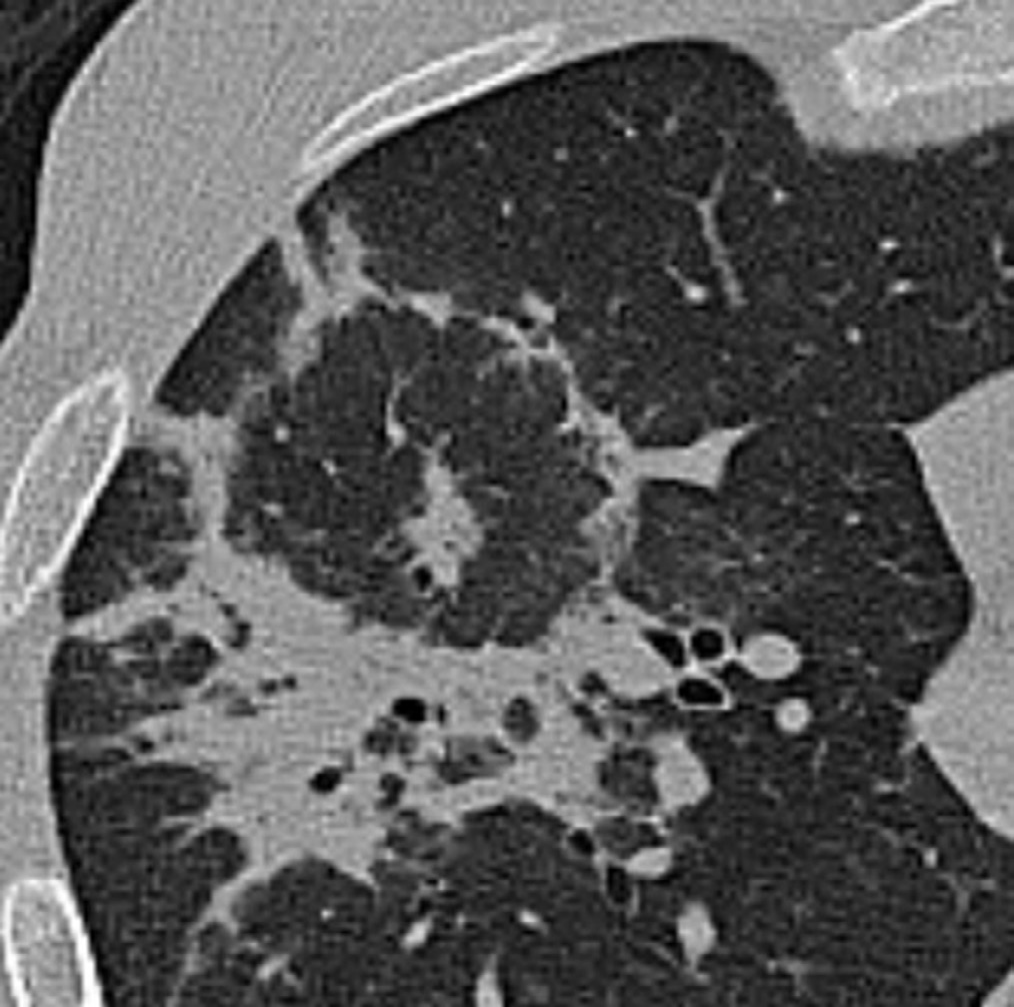
The “bullseye sign” in a 44-year-old male COVID-19 patient (Omicron variant) with fever and dry cough for 7 days.

Considered as a variant of reverse halo sign, this pulmonary feature consists of central high attenuation focus surrounded by an inner ring of air and an outer complete or incomplete ring-like consolidation.^8^ The central nodule is centrilobular, the outer ring of ground glass perilobular, while the inner ring of air corresponds to sparing of the remainder lobule (Figures 5 and 6).

### Tree-in-bud appearance

**Figure 7.**
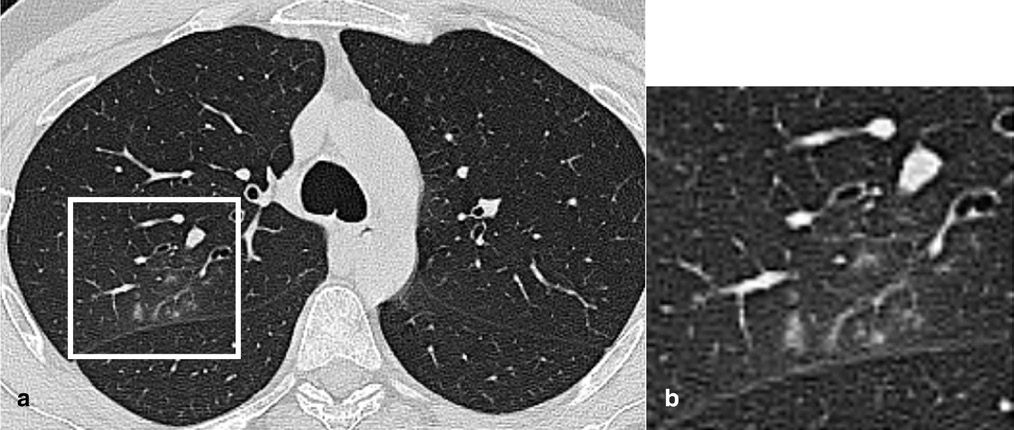
Axial CT image of a 25-year-old male Covid-19 patient (γ variant) showed perifissural small centrilobular nodules connected to linear branching opacities (white box), in right upper lobe.

**Figure 8.**
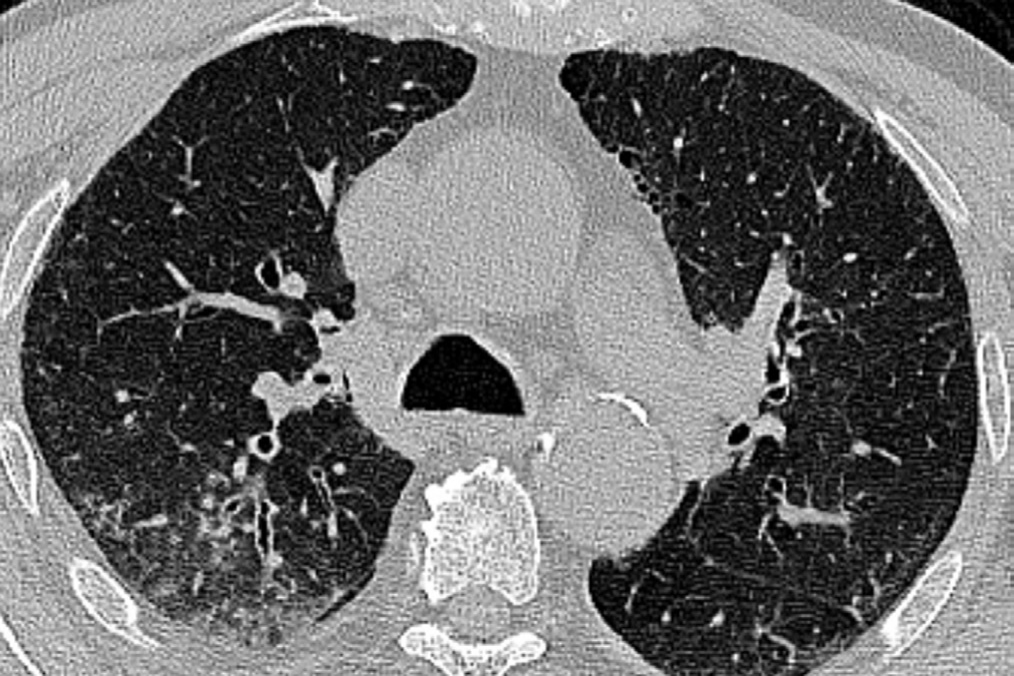
Axial CT image of a 78-year-old male Covid-19 patient (α variant), showed tree-in-bud opacities in right upper lobe with bronchial wall thickening.

Tree-in-bud appearance consists of centrilobular nodules connected to multiple branching linear structures, most observed in lung’s periphery (Figures 7 and 8). Originally described in cases of endobronchial Tubercolosis, this pattern is now considered as a CT feature of many differents infectious agents.^9^

### Diffuse GGO with centrilobular distribution

**Figure 9.**
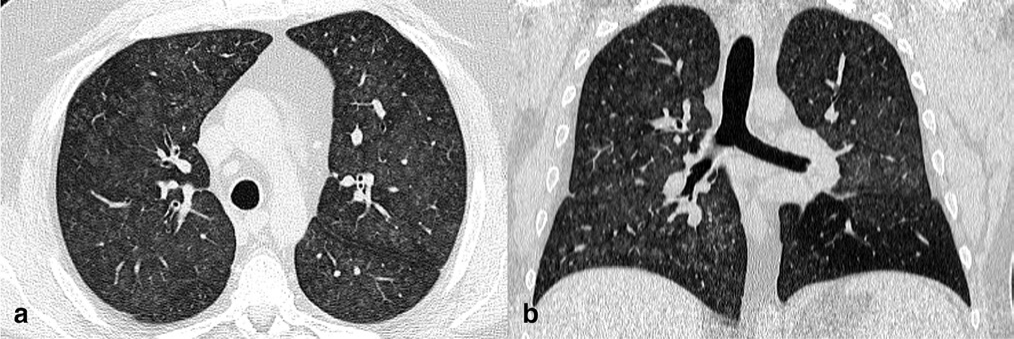
CT images of a 58-year-old male patient with confirmed COVID-19 pneumonia (γ variant). Axial (a) and coronal reformatted (b) images showed diffuse lung involvement with ill-defined centrilobular GGO and subpleural sparing. GGO, ground-glass opacity.

**Figure 10.**
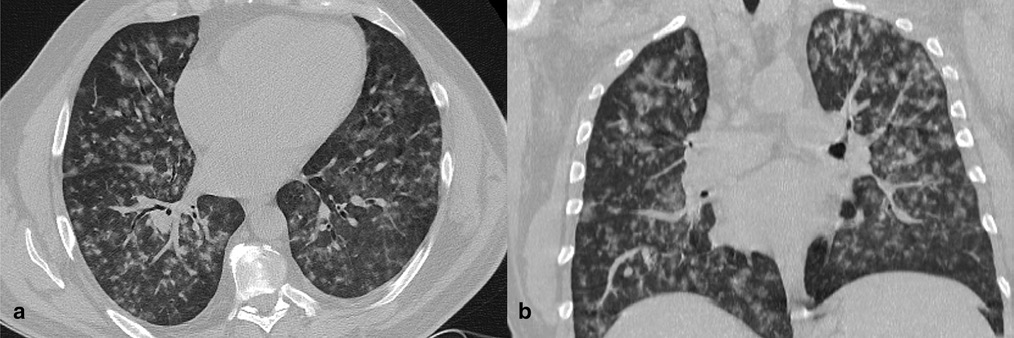
A 42-year-old female COVID-19 patient (γ variant) presenting fever and chills for 4 days. Axial (a) and coronal reformatted (b) images showed bilateral ill-defined centrilobular GGO. GGO, ground-glass opacity.

Variants show also diffuse symmetric, ill-defined GGO in both lung with centrilobular predominant distribution (Figures 9 and 10); this imaging pattern overlaps with other viral infections, such as CMV pneumonia.^10^

### White lung

**Figure 11.**
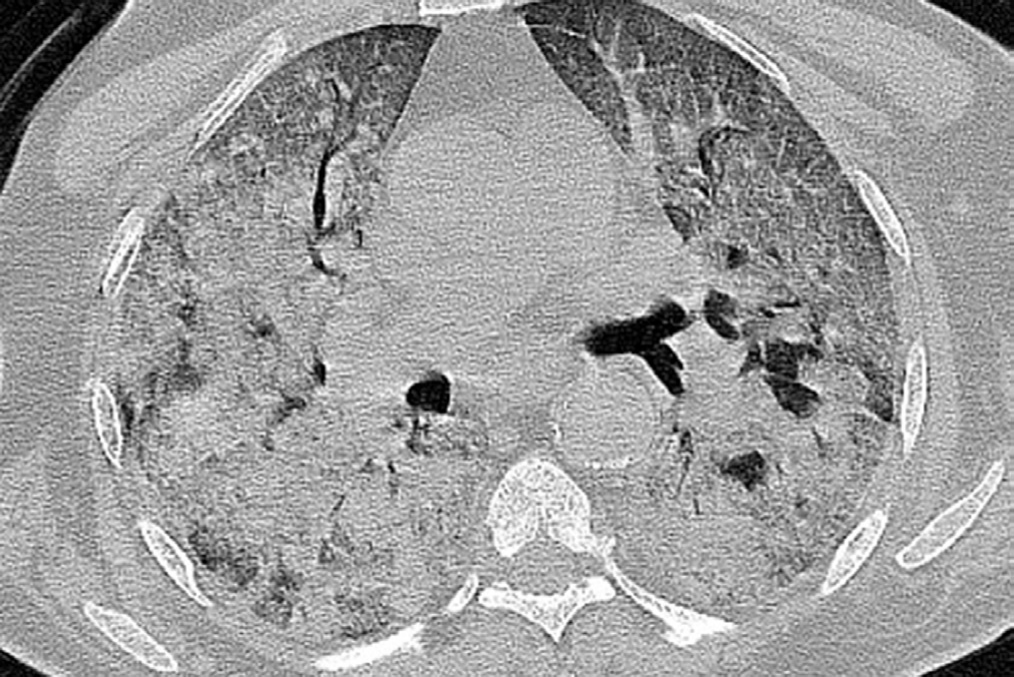
Axial CT image of a 68-year-old patient with COVID-19 pneumonia (δ variant), with symptoms for 4 days, showed bilateral extensive consolidations. The patient died 5 days later.

**Figure 12.**
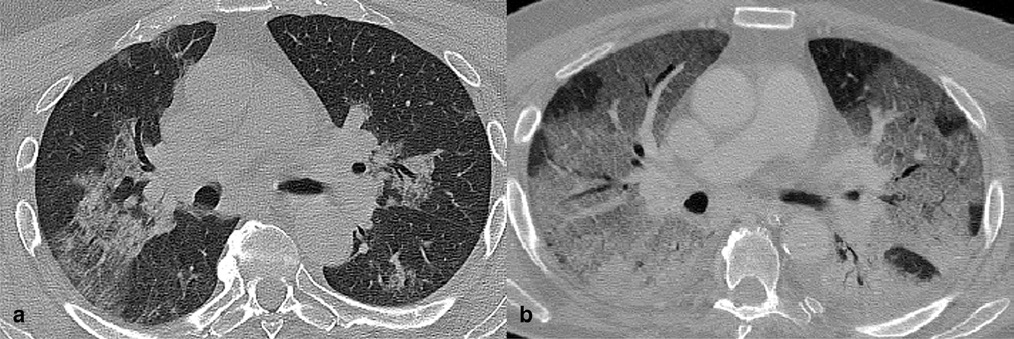
Axial CT image slices of a female COVID-19 patient (α Variant) of 81-year-old. a) CT scan, performed after 1 day from symptoms onset, showed a mixed pattern of GGO and consolidation in upper lobes with peribronchovascular involvement. b) CT obtained after 5 days from symtoms onset. Lesions increased with higher density and superimposed thickening of the interlobular septum in both lungs, with predominant dorsal consolidations.

As pulmonary impairment gets worse, GGOs increase, spreading to more lobes and merging each other, with inter- and intralobular septal reticular thickening and partial consolidation. In patients with complicated pneumonia, dense consolidation involving all lobes become prevalent. This pattern, called “white lung”, can also be observed in other lung diseases (viral infection, drug toxicity). It represents the CT correlate of the underlying pathophysiology of pneumonia as it progresses to acute respiratory distress syndrome (ARDS), predicting a worse outcome.^10^ (Figures 11 and 12)

### Cavitation

**Figure 13.**
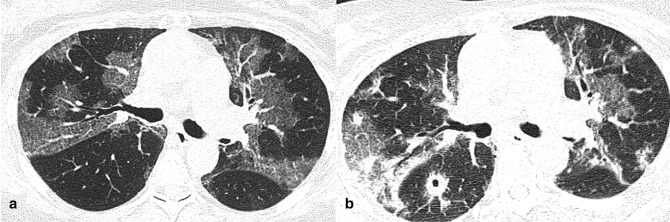
Evolution of CT findings in a 56-year-old female COVID-19 patient (δ variant). (a) CT scan, performed after 3 days from symptom onset, showed GGO with superimposed inter- and intralobular septal thickening (crazy-paving pattern) in upper lobes. (b) Scan obtained on day 7 from symptom onset, showed peripheral consolidations. In apical segment of right lower lobe, CT demonstrated rounded consolidation with cavitation in context.

Cavitation is a circumscribed abnormal gas-filled space of the lung, seen as a low-density area within a pulmonary consolidation, a mass, a nodule. Salehi et al showed that cavitation represents the evolution of lung consolidation, visible on CT in later stage of Covid-19 pneumonia^11^ (Figure 13).

### Pericardial effusion

**Figure 14.**
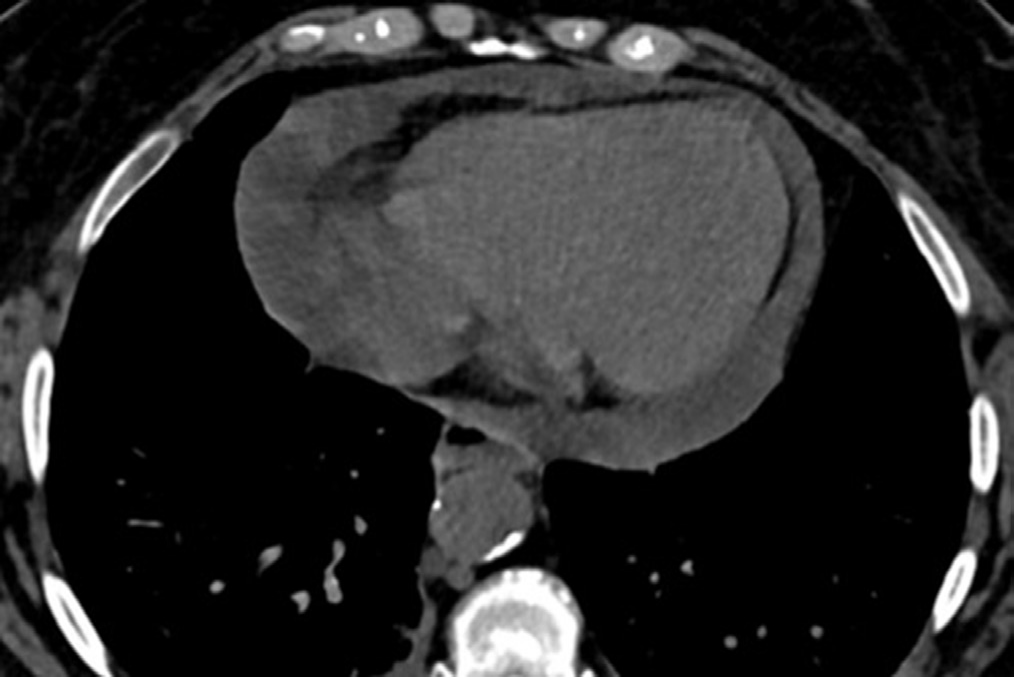
A 53-year-old female COVID-19 patient (Omicron variant), presenting with dry cough, dyspnea and fever up to 39° for 3 days. Axial CT image showed large amount of pericardial effusion.

Several studies stated that pericardial effusion is a rare finding in Covid-19 patients (5% of total population infected with wild type), especially in the later stage of pneumonia, representing a sign of disease progression.^11,12^ Patients infected with variants frequently showed this feature from the earliest stage of disease (Figure 14).

## Conclusions

This pictorial assay summarizes main chest radiological features identified in a wide range of spectrum of manifestation, in patients infected with Covid-19 variants, highlighting differences compared to wild type virus. In our routine screening CT for diagnosis in emergency department of Policlinico Umberto I, we reported atypical chest findings, non-specific or uncommon for Covid-19 pneumonia, potentially attributable to variants, that could be observed in a large number of conditions, leading to diagnostic difficulty.

The imaging hallmark of Covid-19 pneumonia, described in many publications, is a bilateral confluent and extensive GGO and patchy consolidation with prevalent subpleural distribution and lower lobes predominance, mostly posterior basal segments. Inter- and intralobular septal thickening may be present in more advanced cases (crazy paving).

Covid-19 variants produce different chest CT pattern, characterized by GGO with random distribution (both peripheral and central interest) and mainly upper lobe involvement; we reported single isolated GGO or lobar consolidation, thee-in-bud pattern, targeted pulmonary sign.

In the most critical cases, CT scan showed widespread consolidation involving all lobes (white lung).

Furthermore cavitation and pericardial effusion, quite rare findings during first wave of pandemic, were frequently observed with variants.

Diagnostic imaging of COVID has its own limitations. Imaging profile of Covid-19 infection is composite and constantly evolving, with a wide range of CT features, as well as clinical manifestations.

Our pictorial essay displays imaging findings of variants, to help radiologists in their detection to speed-up diagnostic workflow in symptomatic patients; these CT manifestations are not pathognomonic and are observed in other lung infectious and non-infectious processes; a lack of their detection may lead to misdiagnosis and delay in the isolation of Sars-CoV-2 patients.

Those limitations due to aspecific and variable CT features could be partially overcome by correlating them with clinical history.

The findings described and main differential diagnoses are summarized in Table 1.

**Table 1.** CT features in patients infected with variants and main differentials diagnoses

Features associated with variants	Differential diagnoses
GGO with prevalent upper lobes involvement and random distribution	Pulmonary hemorrhage Granulomatous diseases (Sarcoidosis) Acute pulmonary edema
Single isolated involvement	Primary lung cancer (adenocarcinoma) AAH Inflammatory opacity
Isolated lobar consolidation	Lobar pneumonia (*S. pneumoniae, K. pneumoniae, L. pneumophila*) Organizing pneumonia Primary lung cancer
Pulmonary target sign (bullseye sign)	Organizing pneumonia
Tree in bud appearance	Infectious bronchiolitis Viral pneumonia (RSV, HPIV) Bacterial pneumonia (*M. pneumoniae, H. influenza*, MTB, NTM) Fungal pneumonia (Aspergillus) Non-infectious bronchiolitis Hipersensitivity pneumonia Smoking-related lung disease (RB-ILD) Follicular bronchiolitis
Diffuse GGO with centrilobular distribution	Infectious diseases Viral pneumonia (CMV, H1N1) Fungal pneumonia (Pneumocystis Jirovecii) Pulmonary hemorrhage AIP
White lung	Viral pneumonia Drug-induced lung disease Acute pulmonary edema
Cavitation	Infectious diseases Septic embolism Bacterial pneumonia (*S. aureus, S.pneumoniae*, MTB, NTM, Nocardia) Fungal pneumonia Primary lung cancer Lung metastases Vasculitis
Pericardial effusion	Infectious pericarditis (H1N1, ADV, MTB) Hemopericardium

AAH, atypical adenomatous hyperplasia; ADV: adenovirus;AIP, acute interstitial pneumonia; CMV: cytomegalovirus; GGO, ground-glass opacity; HPIV: human para-influenza virus; MTB: Mycobacterium tubercolosis; NTM: non-tuberculous mycobacteria; RB-ILD: respiratory bronchiolitis interstitial lung disease; RSV: respiratory syncytial virus.

H1N1: subtype of Influenza A virus
